# Patterns and outcomes of patients with abdominal injury: a multicenter study from Iran

**DOI:** 10.1186/s12873-024-01002-0

**Published:** 2024-05-31

**Authors:** Sara Mirzamohamadi, Mohammad Navid HajiAbbasi, Vali Baigi, Payman Salamati, Vafa Rahimi-Movaghar, Mohammadreza Zafarghandi, Mehdi Nasr Isfahani, Esmaeil Fakharian, Seyed Houssein Saeed-Banadaky, Morteza Hemmat, Akram Zolfaghari Sadrabad, Salman Daliri, Sobhan Pourmasjedi, Seyed Mohammad Piri, Khatereh Naghdi, Seyed Amir Miratashi Yazdi

**Affiliations:** 1 Sina Trauma and Surgery Research Center, Tehran University of Medical Science, Tehran, 1136746911 Iran; 2 Isfahan University of Medical Sciences, Isfahan, Iran; 3 Department of Epidemiology and Biostatics, School of Public Health, Tehran University of Medical Sciences, Tehran, Iran; 4 Trauma Data Registration Center, Isfahan University of Medical Sciences, Isfahan, Iran; 5 Trauma Research Center, Kashan University of Medical Sciences, Kashan, Iran; 6 Trauma Research Center, School of Medicine, Rahnemoon Hospital, Shahid Sadoughi University of Medical Sciences, Yazd, Iran; 7 Saveh University of Medical Sciences, Saveh, Iran; 8 Clinical Research Development Center, Imam Reza Hospital, Kermanshah University of Medical Sciences, Kermanshah, Iran; 9 Clinical Research Development Unit, Imam Hossein Hospital, Shahroud University of Medical Sciences, Shahroud, Iran

**Keywords:** Abdominal injury, Blunt, Penetrating

## Abstract

**Background:**

Injury is one of the leading causes of death worldwide, and the abdomen is the most common area of trauma after the head and extremities. Abdominal injury is often divided into two categories: blunt and penetrating injuries. This study aims to determine the epidemiological and clinical characteristics of these two types of abdominal injuries in patients registered with the National Trauma Registry of Iran (NTRI).

**Methods:**

This multicenter cross-sectional study was conducted with data from the NTRI from July 24, 2016, to May 21, 2023. All abdominal trauma patients defined by the International Classification of Diseases; 10th Revision (ICD-10) codes were enrolled in this study. The inclusion criteria were one of the following: hospital length of stay (LOS) of more than 24 h, fatal injuries, and trauma patients transferred from the ICU of other hospitals.

**Results:**

Among 532 patients with abdominal injuries, 420 (78.9%) had a blunt injury, and 435 (81.7%) of the victims were men. The most injured organs in blunt trauma were the spleen, with 200 (47.6%) and the liver, with 171 (40.7%) cases, respectively. Also, the colon and small intestine, with 42 (37.5%) cases, had the highest number of injuries in penetrating injuries. Blood was transfused in 103 (23.5%) of blunt injured victims and 17 (15.2%) of penetrating traumas (*p* = 0.03). ICU admission was significantly varied between the two groups, with 266 (63.6%) patients in the blunt group and 47 (42%) in penetrating (*p* < 0.001). Negative laparotomies were 21 (28%) in penetrating trauma and only 11 (7.7%) in blunt group (*p* < 0.001). In the multiple logistic regression model after adjusting, ISS ≥ 16 increased the chance of ICU admission 3.13 times relative to the ISS 1–8 [OR: 3.13, 95% CI (1.56 to 6.28), *P* = 0.001]. Another predictor was NOM, which increased ICU chance 1.75 times more than OM [OR: 1.75, 95% CI (1.17 to 2.61), *p* = 0.006]. Additionally, GCS 3–8 had 5.43 times more ICU admission odds than the GCS 13–15 [OR:5.43, 95%CI (1.81 to 16.25), *P* = 0.002] respectively.

**Conclusion:**

This study found that the liver and spleen are mostly damaged in blunt injuries. Also, in most cases of penetrating injuries, the colon and small intestine had the highest frequency of injuries compared to other organs. Blunt abdominal injuries caused more blood transfusions and ICU admissions. Higher ISS, lower GCS, and NOM were predictors of ICU admission in abdominal injury victims.

## Introduction

Trauma is one of the leading causes of death worldwide [1–3] and the leading cause of death in people under 44 years old [4, 5]. According to the WHO report, in 2019, about 4.4 million deaths due to injuries were recorded, which includes 8% of all causes of death. Among the causes of death due to injuries, road accidents, drowning, falls, burns, and violence against oneself and others are pointed out [6]. Also, 14,000 deaths due to injury are recorded daily, expected to increase by 40% in 2030 [7].

One-fifth of injury mortalities are caused by severe abdominal injuries [8]. Also, the highest prevalence of abdominal injury occurs between the ages of 20 and 40, dramatically impacting the workforce and society’s economy [8]. The abdomen is the third most common region of ​​the body after the head and extremity that suffered from trauma. Around the world, the mortality rate due to abdominal injury is reported between 1 and 20%. Also, in the study of Wiik Larsen J et al., the prevalence of abdominal injury was reported as 7.2 per 100,000 people [3].

In the United States (US) and Korea, road-related accidents (including bicycle, pedestrian, motorcycle) were the leading cause of blunt abdominal injury [9, 10]. Other causes include falls, sports injuries, and industrial accidents. Blunt abdominal injury can cause damage to internal organs and internal bleeding. The liver, spleen, and intestine are the most common organs affected by this type of injury, and due to the indirect nature of this injury, diagnosis is difficult and often time-consuming. Although the outcome of patients with blunt abdominal injury has improved in the last two decades, in patients with multiple organ injuries, the in-hospital mortality rate was reported as 3–10% [10]. Also, according to reports, about 90% of abdominal injuries are blunt [5, 9, 11]. Penetrating abdominal injury is usually caused by stabs and gunshots; most organs damaged in this type include the small intestine, large intestine, liver, and intra-abdominal vessels. Penetrating abdominal trauma accounts for 35% of referrals to urban trauma centers in the US [4]. In Turkey’s studies as a Middle Eastern country, the mortality rate of abdominal trauma was reported at 10.1–19.4% [11].

This study aims to compare epidemiological characteristics and clinical outcomes between blunt and penetrating abdominal injuries in the National Trauma Registry of Iran (NTRI).

## Methods

### Study design

NTRI is a hospital-based registry launched in 2016 at Sina Hospital [12, 13], Tehran, that includes 24 trauma centers. This cross-sectional study was conducted with NTRI data from July 24, 2016, to May 21, 2023, in Sina Hospital of Tehran, Imam Hossein Hospital of Shahroud, Shahid Rahnemoon Hospital of Yazd, Shahid Modarres Hospital of Saveh, Imam Khomeini Hospital of Urmia, Al-Zahra Hospital of Isfahan, Shahid Beheshti Hospital of Kashan, and Taleghani Hospital of Kermanshah.

### Study population

All patients with abdomen injuries defined by the diagnostic International Classification of Diseases, 10th Revision (ICD-10) code admitted to trauma registry member hospitals with one of the following criteria included: hospital length of stay (LOS) more than 24 h, fatal injuries, and trauma patients transferred from the ICU of other hospitals. The patients who were excluded from the study did not have abdominal organ damage and were discharged after examination and imaging or had a laceration in the abdominal wall that did not pass through the peritoneum, and the wound was closed.

### Data collection

The NTRI included 109 variables that two registered nurses completed through interviews with patients and the hospital information system at each trauma hospital. This data is sent to physicians in an electronic system for quality review. In this study, we used the following variables: gender, age, education, cause of injury, injury severity score (ISS), hospitalization in an intensive care unit (ICU), length of stay (LOS), organ injured, multiple or single trauma, pulse rate on arrival, systolic blood pressure on arrival, treatment, death, and blood transfusion. Demographic data, including gender and age, were collected from the patient records. Education level and cause of injury were collected from patient review by a nurse, and a physician measured vital signs, including systolic blood pressure and pulse rate, during the visiting time at the emergency room. A nurse collected LOS, ICU admission, blood transfusion, injured organ, treatment, and death in the hospital from patients’ documents.

This study divided the abdomen injury into two groups: blunt and penetrating. Penetrating injuries are every injury crossing the peritoneum and penetrating the abdomen cavity. Blunt injuries had several causes, including road traffic incidents (RTI), falls, and forces. RTI included injuries to pedestrians, bikers, car occupants, bicycles, and heavy vehicle accidents. Penetrating causes of injuries included stabs/cuts and firearms (shotgun and gunshot).

The sum of squared AIS calculates ISS for the three most severe injuries in each part of the body. This study categorized ISS as 1–8, 9–15, and ≥ 16 [14]. Patients entered one of these groups according to their education years and degrees: no formal education (0 years), primary (1–5 years), secondary (6–12 years), and higher education with a university degree. Hypovolemia criteria included SBP < 90 and/or pulse rate above 120. Also, we calculated the shock index based on heart rate divided by SBP. Patients with a shock index of more than one are considered to have hypovolemic shock. Patient treatment is divided into operative management (OM) and non-operative management (NOM). Number of injuries included two groups: Multiple trauma (MT) considered as other body part injuries in addition to the abdomen injury, only abdomen injury.

### Statistical analysis

The nominal and categorical variables were presented as counts and percentages. Also, continuous variables with normal distribution were described by mean ± standard deviation (SD). The chi-square test was used to compare nominal or categorical variables, and the independent t-test was used to compare continuous variables between blunt and penetrating injuries. *P*-value < 0.05 accepted statistical significance in all the tests. Data analysis was done using STATA 14.

## Results

**Fig. 1:**
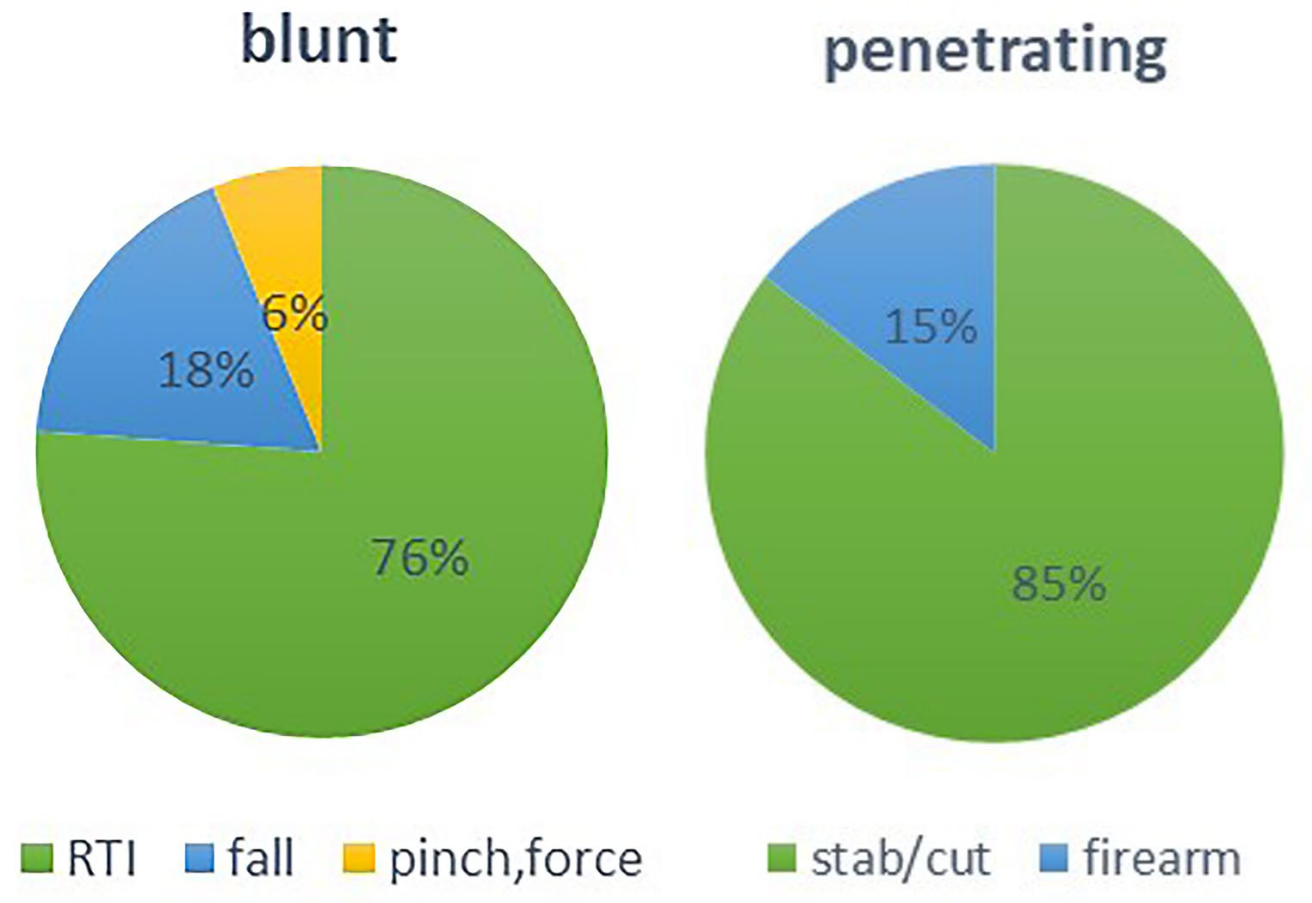
Causes of injury in blunt and penetrating abdominal trauma RTI: Road Traffic Injury

Of the 50,000 patients registered in the NTRI between 2016 and 2023, 1,181 patients complained of an abdominal injury. Of 532 patients in our study, 435 (81.7%) were men, and 97 (18.2%) were women. Four- hundred twenty (78.9%) patients had the blunt, and 112 (21.0%) had the penetrating injury. In both injury types, most of the victims were men. The mean (SD) of age was 30.9 (SD = 17.6) in the blunt group and 31.6 (SD = 13.1) in the penetrating group. Age distribution between the two groups had statistically significant differences. The age group 21 to 40 in blunt group was 173 (41.2%) vs. 69 (61.6%) in penetrating (*p* = 0.001). RTI was the cause of injury in most blunt injuries, with 319 (76%) cases, while stab/cut wounds, with 94 (83.9%) cases, were the leading cause of penetrating injuries significantly (*p* < 0.001)—figure 1. The baseline and clinical characteristics of patients compared between blunt and penetrating abdominal injury are shown in Table 1.

**Table 1:** Baseline characteristics of patients with abdominal injury, N (%)

Variables	Blunt *N* = 420	Penetrating *N* = 112	*p*-value
Gender			0.07
Male	337 (80.2)	98 (87.5)	
Female	83 (19.8)	14 (12.5)	
Age, Mean (SD), year	30.9 (17.6)	31.6 (13.1)	0.68
Age group			**0.001**
1 to 20	133 (31.7)	22 (19.6)	
21 to 40	**173 (41.2)**	**69 (61.6)** ^*^	
> 40	114 (27.1)	21 (18.8)	
Educational level			0.243
Illiterate	15 (3.6)	3 (2.7)	
Primary	281 (67.7)	84 (75)	
Secondary	52 (12.5)	13 (11.6)	
Diploma	51 (12.3)	12 (10.7)	
University degree	16 (3.9)	0 (0)	

^*^Bolded numbers mean these N (%) are significantly different between the two groups

Two hundred sixty-six (63.3%) patients with blunt injury had injuries to other parts of the body in addition to the abdomen, while in the penetrating group, most victims, 76 (67.9%), had abdomen injury solely (*p* < 0.001). Chest trauma, with 26 (23.2%) cases, was the most concomitant injury in penetrating injury. In contrast, in blunt injury, head, neck, and face with 113 (27%), chest with 114 (32%), and extremity with 135 (32.2%) cases more than other body parts suffered from abdominal injury. Ten (2.4%) victims in the blunt group and 79 (70.5%) in the penetrating were injured by the assault (*p* < 0.001) (Table 2).

**Table 2:** Characteristics of injury, organ damage, and place of injury in blunt and penetrating abdomen injuries, N (%)

Variables	Blunt *N* = 420	Penetrating *N* = 112	*P*- value
Intentional	10 (2.4)	79 (70.5)	**< 0.001**
Number of injuries			
Multiple trauma	266 (63.3)	36 (32.1)	**< 0.001**
Only abdomen injury	154 (36.7)	76 (67.9)	
Concomitant injuries			
Head, neck, and face	113 (27)	4 (3.6)	< 0.001
Chest	114 (32)	26 (23.2)	0.082
Extremity	135 (32.2)	9 [8]	< 0.001
Pelvic	45 (10.7)	0 (0)	< 0.001
Spine	60 (14.3)	0 (0)	< 0.001
Organ damage			
Liver	171 (40.7)	33 (29.4)	0.57
Spleen	200 (47.6)	21 (18.8)	**< 0.001**
Pancreas	7 (1.7)	1 (0.9)	0.55
Stomach	14 (3.3)	6 (5.4)	0.31
Intestine & colon	50 (11.9)	42 (37.5)	**< 0.001**
Kidney	54 (12.9)	7 (6.3)	0.051
Vessel	3 (0.7)	4 (3.6)	**0.018**

The most damaged organs due to blunt injury were the spleen, with 200 cases (47.6%), and the liver, with 171 patients (40.7%), whereas 42 (37.5%) of the penetrating group had intestine and colon injuries.

Two hundred and five (48.8%) patients in the blunt trauma group and 81 (72.3%) patients in the penetrating group had ISS of 1 to 8, respectively (*p* < 0.001). In the blunt group, 205 (48.8%) patients had ISS 1 to 8, and in the penetrating group, 81 (72.3%) of them had the same ISS(*p* < 0.001). One hundred-three (23.5%) patients with blunt injury and 17 (15.2%) people in the penetrating group had a blood transfusion (*p* < 0.03). Also, 277 (66%) patients with blunt trauma were treated with NOM, and 132 (31.4%) were treated with OM. In the penetrating group, 37 (33%) patients with NOM and 54 (48.2%) with OM (*p* < 0.001). Eleven (7.7%) patients in the blunt and 21 (28%) victims in the penetrating group had negative laparotomy (*p* < 0.001). In-hospital mortality occurred in 21 (5%) blunt-injury victims and 3 (2.7%) patients in the penetrating group. Blunt-injury victims (63.6%) were significantly admitted to the ICU more than penetrating injuries (42%) (*p* < 0.001). LOS of patients with blunt injury was more prolonged than penetrating victims significantly (*p* = 0.001) (Table 3).

**Table 3:** clinical characteristics in blunt and penetrating abdomen injuries, N (%)

Variables	Blunt *N* = 420	Penetrating *N* = 112	*P*- value
Hypovolemia	59 (14)	17 (15.3)	0.73
Shock Index Normal Hypovolemic shock	350 (84.1) 66 (15.9)	99 (89.2) 12 (10.8)	0.229
ISS			**< 0.001**
1 to 8	**205 (48.8)**	**81 (72.3)**	
9 to 15	115 (26.9)	27 (24.1)	
≥ 16	**102 (24.3)**	**4 (3.6)**	
Blood transfusion	103 (23.5)	17 (15.2)	**0.03**
Treatment			**< 0.001**
OM	132 (34)	54 (67)	
NOM	277 (66)	37 (33)	
Laparotomy			**< 0.001**
Positive laparotomy	132 (92.3)	54 (72)	
Negative laparotomy	**11 (7.7)**	**21** (28)	
GCS			0.54
3 to 8	36 (8.6)	6 (5.4)	
9 to 12	33 (7.9)	9 (8.1)	
13 to 15	350 (83.5)	96 (86.5)	
ICU admission	266 (63.6)	47 (42)	**< 0.001**
Mortality	21 (5)	3 (2.7)	0.29
LOS, median (IQR), hrs	179 (215)	134 (120)	**0.001**

ISS: injury severity score; GCS: Glasgow coma scale; ICU: intensive care unit; OM: operative management; NOM: non-operative management; LOS: length of stay

ICU admission. Univariate logistic regression revealed 21-40-year-old patients had a 0.68 times lower chance than the > 40-year-old to ICU admission [OR:0.68, 95% CI (0.44 to 1.05), *p* = 0.09]. Other predictors of ICU admission were blunt injury [OR:2.43, 95% CI (1.59 to 3.72), *p* < 0.001], hypovolemia [OR: 2.15, 95%CI (1.24 to 3.7), *p* = 0.001], MT [OR:1.19, 95% CI (1.34 to 2.72), *p* < 0.001], NOM [OR:1.87, 95%CI (1.31 to 2.66), *p* < 0.001], GCS 9–12 relative to the 13–15 [OR:4.14,95% CI (1.79 to 9.54), *p* = 0.006] and GCS 3–8 relative to the 13–15 [OR:8.1, 95%CI (2.84 to 23.08), *p* < 0.001], ISS 9–15 relative to the 1–8 [OR:1.72, 95% CI (1.14 to 2.61), *p* < 0.001] and ISS ≥ 16 relative to the 1–8 [OR:5.08, 95%CI (2.9 to 8.88), *p* < 0.001].

In the multiple logistic regression model after adjusting, ISS ≥ 16 increased the chance of ICU admission 3.13 times, and ISS 9–15 increased 1.79 times relative to the ISS 1–8 [OR: 3.13, 95% CI (1.56 to 6.28), *p* = 0.001], [OR:1.79, 95% CI (1.05 to 3.04), *p* = 0.03]. Another predictor was NOM, which increased ICU chance 1.75 times more than OM [OR: 1.75, 95% CI (1.17 to 2.61), *p* = 0.006]. Alao, GCS 9–12 had 3.36 times more odds of ICU admission, and GCS 3–8 had 5.43 times more odds compared to the GCS 13–15 [OR:3.36, 95% CI (1.34 to 8.37), *p* = 0.009], [OR:5.43, 95%CI (1.81 to 16.25), *p* = 0.002] respectively—Table 4.

**Table 4:** odds ratio (OR) and 95% confidence interval (CI) of ICU admission

ICU admission	Univariate OR (95% CI)	adjusted OR (95% CI)	*P*-value
Age group			
> 40	References		
21 to 40 1 to 20	0.68 (0.44 to 1.05) 1.38 (0.85 to 2.25)	0.76 (0.48 to 1.22) 1.37 (0.80 to 2.33)	0.26 0.24
Penetrating trauma	References		0.055
Blunt trauma	2.43 (1.59 to 3.72)	1.62 (0.98 to 2.66)	
Hypovolemia	2.15 (1.24 to 3.7)	1.57 (0.83 to 2.97)	0.15
M.T	1.91 (1.34 to 2.72)	0.92 (0.56 to 1.53)	0.77
ISS			
1 to 8	References		
9 to 15 ≥ 16	1.72 (1.14 to 2.61) 5.08 (2.9 to 8.88)	1.79 (1.05 to 3.04) 3.13 (1.56 to 6.28)	**0.03** **0.001**
GCS			
13 to 15	References		
9 to 12 3 to 8	4.14 (1.79 to 9.54) 8.1 (2.84 to 23.08)	3.36 (1.34 to 8.37) 5.43 (1.81 to 16.25)	**0.009** **0.002**
Treatment			
OM	References		
NOM	1.87 (1.31 to 2.66)	1.75 (1.17 to 2.61)	**0.006**

OM: operative management; NOM: non-operative management; ISS: injury severity score; GCS: Glasco coma scale; ICU: intensive care unit; M.T: multiple trauma

Blood transfusion. Univariate logistic regression revealed blunt trauma increased the chance of blood transfusion 1.83 times more than penetrating [OR: 1.83, 95% CI (1.04 to 3.22), *p* = 0.034]. Also, MT with 5.2 OR [OR: 5.2, 95% CI (3.09 to 8.72), *p* < 0.001], hypovolemia with 2.27 more odds [OR: 2.27, 95% CI (1.35 to 3.82), *p* = 0.002], and shock index more than one with 2.02 OR [OR: 2.02, 95% CI (1.2 to 3.4), *p* = 0.008] increased blood transfusion chance. Other predictors were ISS 9–15 [OR: 4.75, 95%CI (2.81 to 8.02), *p* < 0.001], and ≥ 16 [ OR: 6.56, 95% CI (3.79 to 11.38), *p* < 0.001] compared to ISS 1–8. In addition, patients who were candidates for OM had a 1.65 times more chance for blood transfusion than the NOM [OR: 1.65, 95% CI (1.09 to 2.49), *p* = 0.016], respectively.

In the multiple logistic regression after adjustment, ISS ≥ 16 increased the chance of ICU admission 3.53 times, and ISS 9–15 increased three times relative to the ISS 1–8 [OR: 3.53, 95% CI (1.79 to 6.96), *p* = 0.001], [OR: 3, 95% CI (1.6 to 5.62), *p* < 0.001]. Other predictors were M.T with 2.28 OR [OR: 2.28, 95% CI (1.17 to 4.43), *p* = 0.015], and OM with 1.89 OR relative to the NOM [OR: 1.89, 95% CI (1.19 to 2.98), *p* = 0.006]—Table 5.

**Table 5:** Odds ratio (OR) and 95% confidence interval (CI) of blood transfusion

Blood transfusion	Univariate OR (95% CI)	adjusted OR (95% CI)	*P*-value
Type of trauma			
Penetrating	Reference	Reference	0.403
Blunt	1.83 (1.04 to 3.22)	1.31 (0.69 to 2.51)	
ISS			
1 to 8	Reference	Reference	
9 to 15 ≥ 16	4.75 (2.81 to 8.02) 6.56 (3.79 to 11.38)	3.00 (1.6 to 5.62) 3.53 (1.79 to 6.96)	**0.001** **< 0.001**
M.T	5.2 (3.09 to 8.72)	2.28 (1.17 to 4.43)	**0.015**
Treatment			**0.006**
NOM OM	Reference 1.65 (1.09 to 2.49)	Reference 1.89 (1.19 to 2.98)	
Hypovolemia	2.27 (1.35 to 3.82)	1.65 (0.76 to 3.6)	0.202
Shock Index			0.767
Normal Hypovolemic shock	Reference 2.02 (1.2 to 3.4)	Reference 1.12 (0.52 to 2.42)	

OM: operative management; NOM: non-operative management; ISS: injury severity score; M.T: multiple trauma

## Discussion

In our study, among 532 patients with an abdomen injury, 78.9% of them were caused by blunt trauma and 76% by RTI. Most victims were men and middle-aged people, like our previous report about gender differences in trauma and other trauma reports [15–17]. Most abdominal injury victims had primary education, as a previous study from NTRI observed primary education as a predominant educational level [18]. Our findings comparing blunt and penetrating abdomen injuries included that blunt injuries had higher ISS, blood transfusion, mortality, and ICU admission rates. Blunt victims managed non-operative in two-thirds of cases. In contrast, penetrating injuries were related to OM and negative laparotomy. The most organ damage was spleen and liver in blunt trauma, similar to other studies [15], and intestine and colon in penetrating injuries. We demonstrated that higher ISS, lower GCS, and NOM were predictors of ICU admission.

At first, Shaftan in 1960 described the observation of abdomen injuries with no significant mortality and morbidity [19]. In our study, we demonstrated blunt injuries were managed non-operative while penetrating victims were managed operative mainly. In cases where patients with blunt injuries are stable, studies have shown that non-operative management is favored as the primary form of treatment. While in penetrating injuries, OM and NOM could be performed. NOM is increasing because of decreasing LOS, hospital costs, and negative laparotomy rates [20, 21], performed in patients with stable hemodynamics without peritonitis signs [22]. Based on the studies, selective NOM in patients with shotgun injuries was better than OM because of better outcomes and lower complications [23]. The study from the USA reported that one-quarter of patients with firearm injuries and one-third of those who suffered from stabs were managed non-operatively. Also, they showed an increase in the NOM rate and a decrease in negative laparotomy [24].

Blunt injury victims had worse trauma outcomes compared to penetrating injuries. Compared to penetrating injuries, they had a higher mortality rate non-significantly and higher LOS, ICU admission, and blood transfusion. In contrast, a study from Germany observed that unstable hemodynamics, mortality rate, and emergency surgery indication were higher in penetrating trauma than in blunt injuries [17]. Another study with a 9.5% mortality rate in abdomen trauma observed that non-survivors suffered from blunt trauma mostly [25]. In the blunt group, ICU admission and LOS were higher than penetrating because of the higher rate of extra abdominal injuries and NOM in this group. Two-thirds of patients with blunt trauma managed non-operatively. In NOM, patients were admitted to the ICU for observation. Besides, these patients had injuries in other body parts besides the abdomen. Multiple trauma patients had a higher chance of ICU admission compared to others. In this line, a study from the Scottland trauma registry showed that patients with injuries in several other body parts besides the abdomen had longer LOS [25]. Blunt victims had non-significantly lower hypovolemia, higher Shock index, and significantly higher blood transfusion compared to the penetrating. However, blunt trauma was not a predictor of blood transfusion; on the contrary, OM, MT, and higher ISS led to blood transfusion.

Blunt trauma had higher ISS than penetrating. ISS ≥ 16 in the blunt group and 1 < ISS < 8 in the penetrating group were more frequent. Based on the studies, higher ISS is associated with a higher ICU admission rate chance [26]. In this line, our blunt victims had higher ISS and more ICU admissions.

In this study, 186 laparotomies were performed for patients from both groups; in total, 17.2% had negative laparotomy, similar to other countries that reported 6 to 25% negative laparotomy [10, 15, 27]. Two-thirds of negative laparotomy belongs to penetrating injuries because of more OM in this group. Eight patients were admitted to ICU after negative laparotomy, and two had a blood transfusion. According to the studies, laparotomy is necessary for patients with hemodynamically unstable, unreliable abdominal examination and abdomen tenderness. There are two recommendations for laparotomy: performing laparotomy earlier in patients with a wound entering the abdomen cavity, whereas newer publications recommend a decision based on the clinical features [27–29]. The first policy indicated that negative laparotomy did not need organ repair. This situation led to some complications, including wound infection, abscess, and organ laceration [27]; therefore, decreasing negative laparotomy is crucial.

Based on the studies, laparotomy, and abdomen organ repair surgery had some complications, including wound infection, abscess, and laceration [27]. These complications may affect patients’ outcomes, for example, longer LOS, ICU admission, blood transfusion, intubation, dialysis or death. On the other hand, the NOM strategy could lead to failure and delayed operation that affected outcomes. Therefore, comparing complications is necessary. Unfortunately, our outcome analysis was performed without considering complications.

Our study had some limitations. Death before arrival at the hospital is not recorded in our trauma registry. Therefore, our mortality rate is confined to hospital stays. Our data did not include other abdominal injury examinations, including diagnostic peritoneal lavage (DPL) and serial examination. Also, Surgical complications after discharge are not recorded in our registry.

## Conclusion

We concluded blunt abdominal injuries had worse outcomes, including ICU admission, LOS, and mortality, compared to penetrating, while there were no worse physiologic signs and symptoms, including GCS and hypovolemia. Because blunt abdominal trauma had more concomitant trauma, had higher ISS than penetrating, and blunt victims were candidates for NOM more than penetrating. Therefore, physicians must pay more attention to blunt victims with normal signs and symptoms. In addition, penetrating victims had a higher rate of negative laparotomy. We recommended developing NOM for penetrating injuries more than current experiences to decrease negative laparotomy and its complications.

## Data Availability

The datasets used and analyzed during the current study are available from Dr. Payman Salamati, director of the NTRI project, on reasonable request.
